# Human Herpesvirus-6 Encephalitis Associated With Acute Necrotizing Encephalopathy in an Immunocompetent Child: A Case Report and Literature Review

**DOI:** 10.7759/cureus.39897

**Published:** 2023-06-03

**Authors:** Esraa AlQasim, Abdulsalam Alawfi, Abdalazeem Hamad, Fouad Alghamdi, Bandar A Albaradi

**Affiliations:** 1 Pediatric Infectious Diseases, King Fahad Specialist Hospital, Dammam, SAU; 2 Pediatric Infectious Diseases, Taibah University, Al-Madinah, SAU; 3 Pediatric Neurology, King Fahad Specialist Hospital, Dammam, SAU

**Keywords:** acute disseminated encephalomyelitis, ganciclovir, foscarnet, acute necrotizing encephalopathy, acute necrotizing encephalitis, encephalitis, human herpesvirus type 6 (hhv-6)

## Abstract

Human herpesvirus type 6 (HHV-6) is a DNA virus considered a member of the Herpesviridae family. HHV-6 is acquired early in life, when it may cause roseola infantum and nonspecific febrile illnesses which is usually a self-limiting disease before the age of two. Primary HHV-6 encephalitis and acute necrotizing encephalopathy (ANE) are rare diseases to occur in immunocompetent children. We describe an unusual case of HHV-6 encephalitis with mixed features of acute necrotizing encephalopathy and acute disseminated encephalomyelitis and contextualize it with a review of the literature on HHV-6 encephalitis in immunocompetent children. Although the incidence of primary HHV-6 encephalitis is rare in immunocompetent children, HHV-6 encephalitis associated with acute necrotizing encephalopathy is a devastating disease, highly fatal and neurologically damaging disease. Therefore, early testing and diagnosis are crucial as well as effective management of encephalitis with antiviral therapy is highly recommended.

## Introduction

Human herpesvirus type 6 (HHV-6) is a DNA virus that belongs to the Herpesviridae family [1]. There are two known types of HHV-6: HHV-6A and HHV-6B. It is well known that HHV-6B is more commonly detected than HHV-6A in peripheral blood mononuclear cells (PBMCs) and cerebrospinal fluid (CSF), and it usually causes a self-limiting primary disease before the age of two, mostly in the form of roseola infantum (the sixth disease). Then, the virus becomes latent [1].

Primary HHV-6 encephalitis is uncommon to occur in healthy children. Immunocompromised patients are more likely to develop encephalitis due to the reactivation of HHV-6, especially after hematopoietic stem cell transplantation [2]. The radiological hallmark of HHV-6 encephalitis is transient abnormal signal intensity in the mesial temporal lobes in magnetic resonance imaging (MRI) with a normal early computed tomography (CT) scan [3]. Positive CSF HHV-6 DNA PCR in symptomatic patients is indicative of active HHV-6 encephalitis, while positive blood real-time PCR is indicative of active systemic infection [1,4]. Acute necrotizing encephalopathy of childhood (ANEC) is an uncommon acute onset of brain damage with a rapid clinical progression that usually affects healthy children after an acute febrile illness, mostly viral illnesses. HHV-6 infection, along with other viruses including influenza A and B viruses and herpes simplex virus, is considered the most common virus predisposing to ANEC. The etiology of ANEC is unknown, although it can be explained by pro-inflammatory cytokines (such as tumor necrosis factor receptor-1, interleukin-1, and interleukin-6), which are produced by overactivation of the immune system in response to acute febrile illness, mostly viral infection. The radiological hallmark of ANEC is multifocal and symmetrical bilateral thalamic lesions [5]. We have multiple treatment modalities for ANEC, including the early use of corticosteroids, plasmapheresis, anticytokine therapies (including tumor necrosis factor-alpha (TNF-α) antagonists and interleukin-6 receptor antagonists), and/or therapeutic hypothermia. All are safe and applicable, but we need more studies to compare them and to detect the best treatment approach [6].

Acute disseminated encephalomyelitis (ADEM) can also be predisposed by viral infections or vaccinations, which will be followed by demyelination of white matter due to acute inflammation. The etiology of this process is also unknown, but it could be related to an abnormal immune response to viral antigen in the central nervous system (CNS). The radiological hallmark of ADEM is asymmetrical involvement of the centrum semiovale, basal ganglia, and thalamus, with or without the "open ring sign". Patients with ADEM usually respond well to high-dose glucocorticoids, intravenous immunoglobulins, and/or plasma exchange [7]. In this report, we describe a patient who has positive HHV-6 encephalitis with clinical and radiological features that are not only typical for HHV-6 encephalitis but also have ANEC features with an ADEM-like picture at the same time. The consent was taken from family members for publication as required.

## Case presentation

A 13-month-old healthy boy was presented to King Fahad Specialist Hospital in Dammam, Saudi Arabia, in July 2019 with fever, runny nose, and dry coughing associated with irritability. Two days later, these symptoms resolved, but he started to have drowsiness and was suddenly unresponsive to external stimuli. After a few hours, he developed two episodes of generalized tonic convulsions associated with bilateral eye up-rolling for less than one minute, which were aborted spontaneously. His family history was unremarkable, and his vaccinations are up-to-date. He was febrile, with a temperature of 39.2°C. Other vital signs were within the normal range. He also has a normal respiratory pattern and negative meningeal signs without abnormal posturing or dysmorphic features. His skin has a petechial rash over the face and chest. His CNS exam revealed a Glasgow Coma Scale of six out of 15, generalized hypertonia, and hyperreflexia with lower limb deep tendon reflexes of +4 and upper limb deep tendon reflexes of +3. In addition, he has positive clonus and positive Babinski signs. However, his pupils were equally reactive, with normal fundoscopic findings and positive gag, cough, and corneal reflexes without any focal neurological abnormality. Of note, other systemic examinations were unremarkable. Laboratory tests showed both normal blood glucose and venous blood gas levels, while CBC revealed mild pancytopenia (hemoglobin was 9.1 g/dL (normal: 10.5-12) and platelet count was 89 × 10^9^/L (normal: 206-553 x 10^9^/L)). His leukocyte count was 3.68 × 10^9^/L (normal: 6 to 17.5) with an absolute neutrophil count of 1.87 × 10^9^/L, which represents 50% of the leukocyte count in the differential count. His CRP was 9 mg/L (normal: < 10). He has a mild elevation in liver enzymes with alanine transaminase (ALT) of 185 unit/L (normal: 7-55) and aspartate aminotransferase (AST) of 93 unit/L (normal: 8-60), but normal serum ammonia and lactate. He also has normal renal functions and electrolytes, as well as a negative urine toxicology screening. The respiratory virus panel and human immunodeficiency virus screening were both negative. Immunological work-up showed no evidence of underlying immunodeficiency.

His cerebrospinal fluid (CSF) sample was taken, and the results revealed that his CSF protein level was significantly elevated at 0.9 g/L (normal range: 0.18-0.45), but all other factors, such as CSF chemistry and hematology, were within normal limits. The bacterial culture of his CSF exhibited no growth, but human herpesvirus type 6 (HHV-6) positive results were found in his CSF neuroviral polymerase chain reaction (PCR) panel. Herpesvirus 6 DNA quantitative PCR was therefore performed on his CSF, and the result revealed a very high quantity of 3,559 copies/mL (reference normal range: ≤ 500 copies/mL).

**Figure 1:**
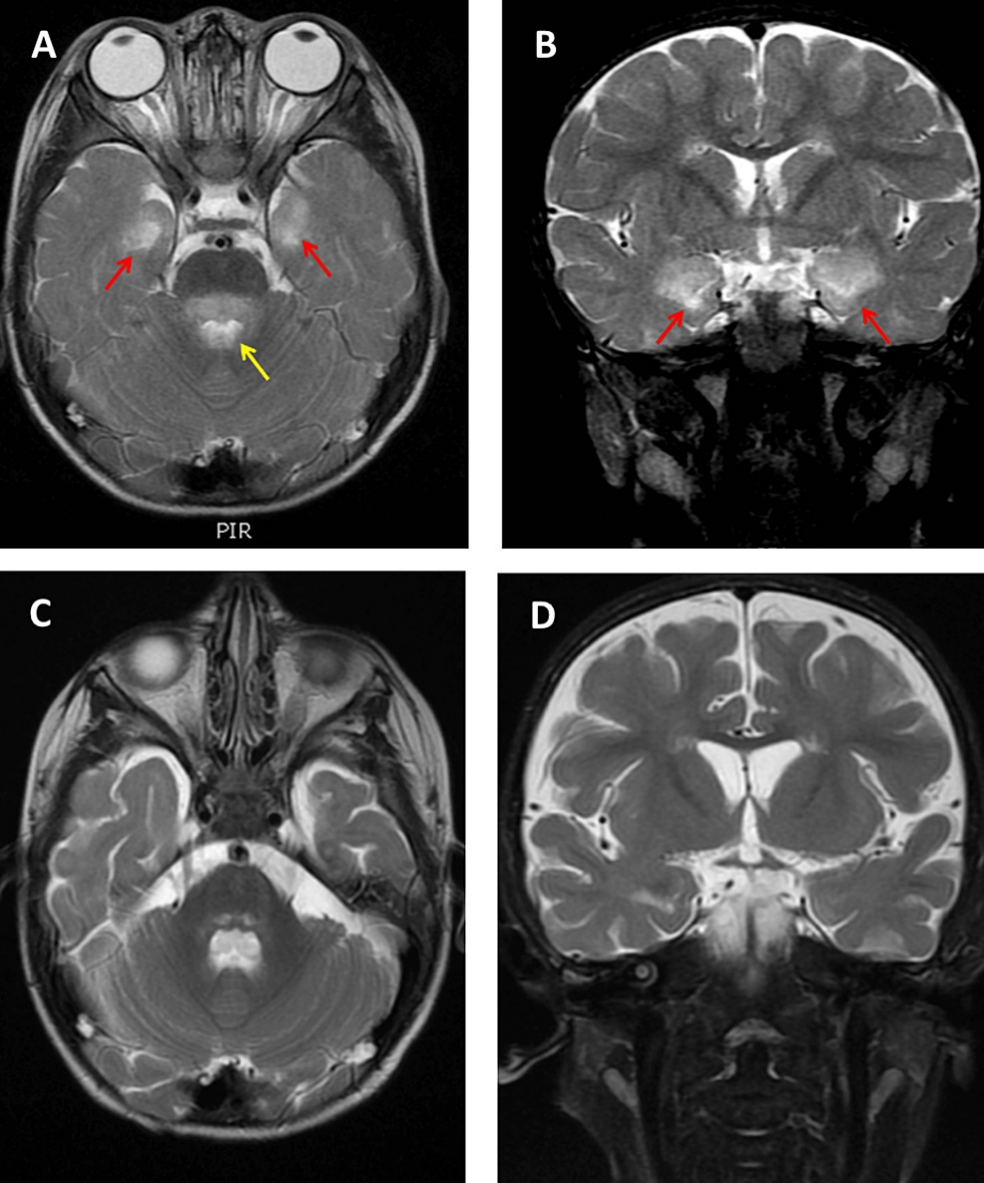
The initial brain MRI images showed hyperintensity in bilateral medial temporal areas The initial brain MRI images showed hyperintensity (red arrows) in bilateral medial temporal areas in axial view (A) and coronal view (B) of T2 modality images, which is very suggestive of herpes simplex virus (HSV) or other Herpesviridae groups of viruses that can cause encephalitis. The "activated reticular system" in the dorsal region of the brainstem (yellow arrow), which is crucial for preserving the patient's degree of consciousness, is also affected. The repeated brain MRI images showed an interval regression of hyperintensity in the bilateral medial temporal area in the axial view (C) image and coronal view (D) image of the T2 modality.

**Figure 2:**
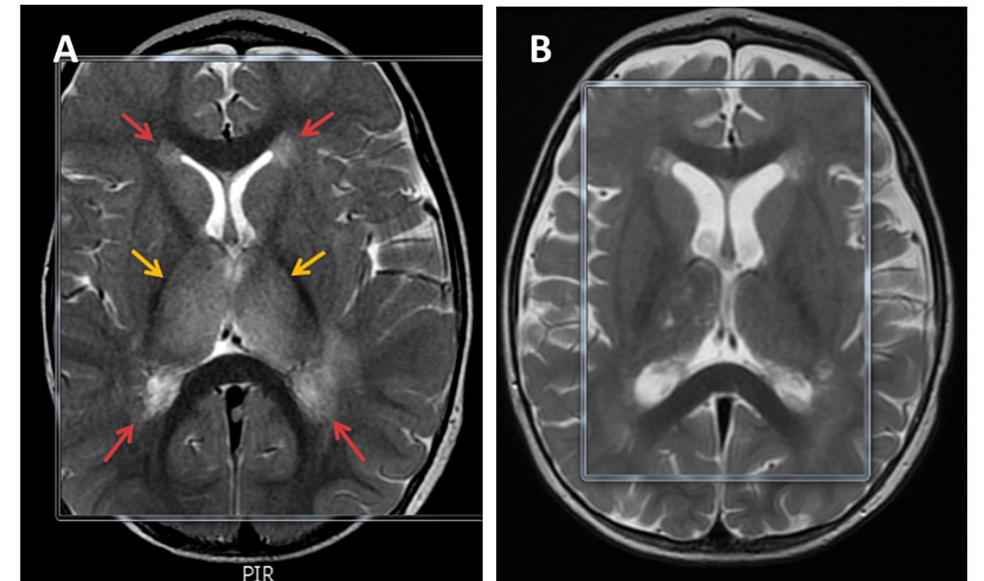
The axial image of the T2 modality The axial image of the T2 modality (A) demonstrates a high signal intensity in the bilateral periventricular area (red arrows) with swollen hyperintensive thalami (yellow arrows), which is a characteristic finding of acute necrotizing encephalopathy of childhood. In the repeated brain MRI images, there was an interval regression of T2 modality hyperintensity in bilateral deep periventricular white matter, with regression of swelling of the thalami and temporal lobes (B). Also, there is a new development of enhancement in bilateral symmetrical anterior temporal horns and lateral ventricles. These areas showed diffusion restriction in the previous study, which likely represents infarcts in the late subacute stage.

**Figure 3:**
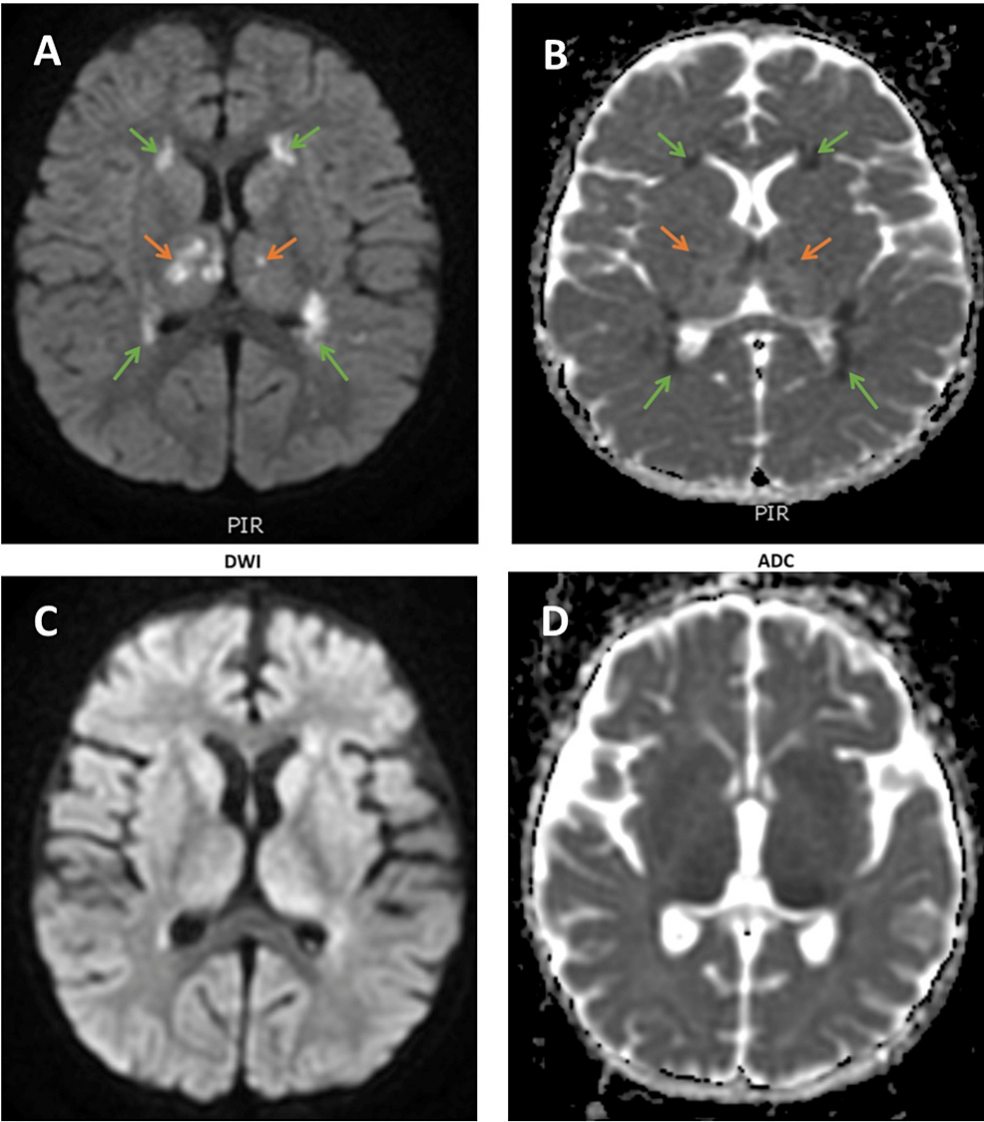
Initial brain MRI images Initial brain MRI images showed symmetrical bilateral periventricular (green arrows) and asymmetrical bilateral thalami (orange arrows) bright spots, which are visible on the axial image of the DWI modality (A). In the ADC modality (B), those same spots appear dark, which indicates limited diffusion and suggests cellular damage. In the repeated brain MRI images, the axial view of both the DWI modality (C) and the ADC modality (D) showed an interval resolution of restricted diffusion regions in bilateral deep periventricular white matter. DWI: diffusion-weighted imaging, ADC: apparent diffusion coefficient.

**Figure 4:**
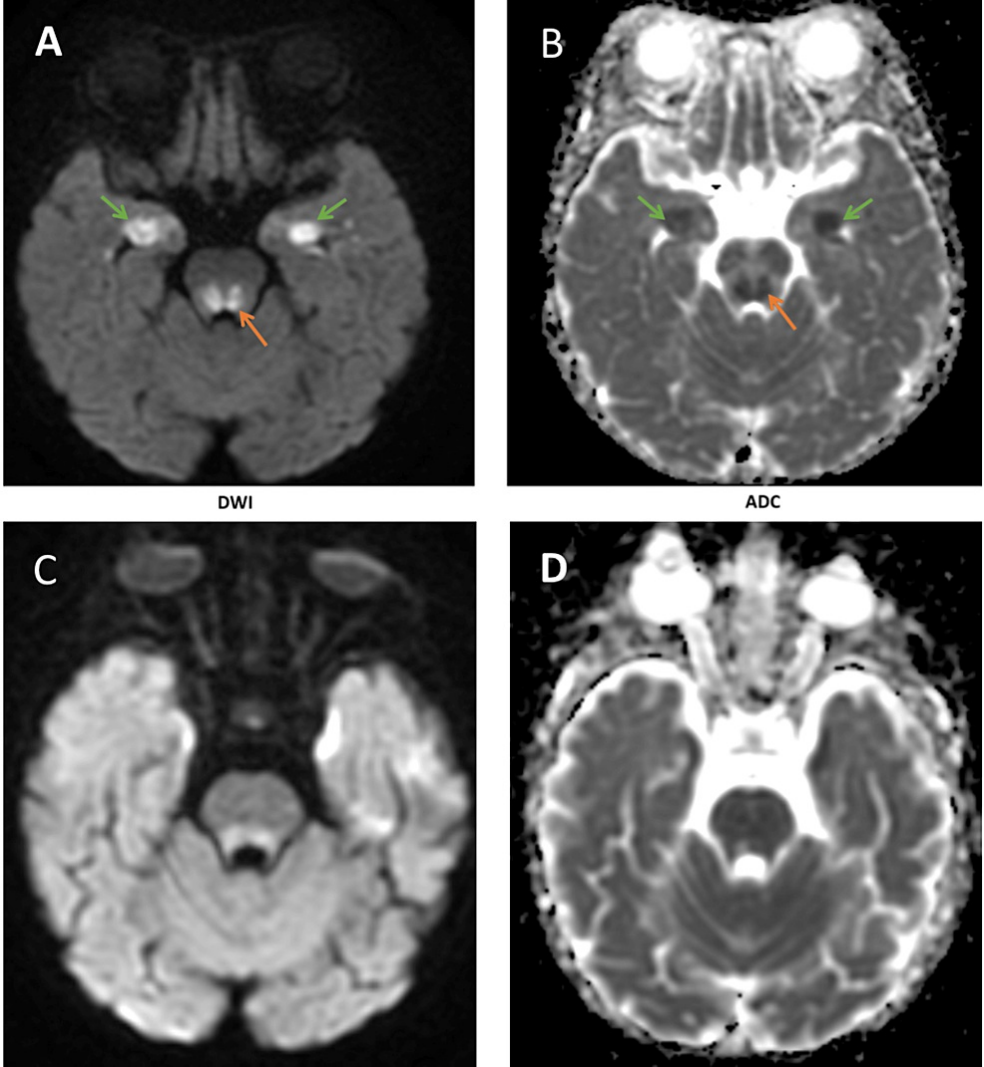
Restricted diffusion in the mesial temporal and dorsal pons Similar to the observation in Figure 3, there is restricted diffusion in the mesial temporal (green arrows) and dorsal pons (orange arrows). The bright spots on the axial DWI modality (A) image appeared dark in the ADC modality (B) image, which eventually resolved in both the DWI modality (C) and ADC modality (D) images. DWI: diffusion-weighted imaging, ADC: apparent diffusion coefficient.

Radiological imaging showed a normal brain CT scan. Brain MRI with contrast showed areas of fluid-attenuated inversion recovery (FLAIR)/T2 hyperintensity and T1 mild hypointensity, which are seen bilaterally in the centrum semiovale, corona radiate, deep periventricular white matter, temporal lobes, thalami, and the medial lemnisci. These lesions show restricted diffusion, do not enhance with contrast, and do not show signs of hemorrhage on the susceptibility-weighted images (see Figures 1-4 ). The basal ganglia, internal capsule, corpus callosum, and corticospinal tracts are spared. The spinal MRI is normal. His electroencephalogram (EEG) showed numerous tonic seizures arising from the left and right temporal areas.

The patient was intubated upon presentation to the emergency room due to his low Glasgow Coma Scale (GCS) and shifted to the pediatric intensive care unit (PICU). He required a small dose of norepinephrine for only one day to keep his blood pressure maintained. During his hospital stay, the patient received intravenous immunoglobulin (IVIG) (one dose over 48 hours), high-dose pulse glucocorticoid (completed five doses), then shifted to gradually tapering prednisolone every week, plasmapheresis (seven sessions), antiepileptic medications (initially phenytoin, then shifted to Keppra and oxcarbazepine), and baclofen with physiotherapy for his hypertonia. Triple antimicrobial agents were commenced empirically, including ceftriaxone, vancomycin, and acyclovir, but shifted to foscarnet and ganciclovir for a total of 21 days when the result of HHV-6 came out.

The patient was extubated after one week of his illness. He started to have spontaneous eye openings after five days of the treatment, but without focusing or concentrating on his surroundings. He lost his ability to walk, sit, swallow, and all his verbal outcomes except moaning. He did not develop any clinical seizures.

After two weeks of his illness, his CSF sample was repeated, with results showing an increase in CSF protein to 4.56 g/L (normal: 0.18-0.45) with persistent positive CSF (HHV-6) PCR. Repeated MRI of the brain revealed interval resolution of restricted diffusion areas with interval regression of FLAIR/T2 hyperintensity in bilateral deep periventricular white matter and regression of swelling of the thalami and temporal lobes. However, there was a new development of enhancement in bilateral symmetrical anterior temporal horns and lateral ventricles. These areas showed diffusion restriction in the previous study, which likely represents infarcts in the late subacute stage.

The patient was discharged from the hospital after one month with the same neurological sequelae. Pediatric neurology, physical therapy, and speech therapy have continued to monitor the patient for more than a three-year period since the onset of his disease. He continued to have epilepsy, sleep disturbances, and sleep myoclonus. Developmentally, his language is currently limited to only three single words, and he gradually started to walk unsupported but with an ataxic gait.

## Discussion

We described a healthy immunocompetent child who had an acute febrile illness that progressed rapidly within two days to acute encephalitis manifested as fever, tonic seizures, a low Glasgow coma scale, and signs of an upper motor neuron lesion. He had elevated CSF proteins and was positive for both CSF and blood PCR for HHV-6B. He has abnormal EEG electrical discharges arising from bilateral temporal areas, and brain MRI showed restriction diffusion in multiple areas that can represent HHV-6 encephalitis, ANEC, and ADEM. This patient has positive CSF HHV-6 PCR with significantly high CSF quantitative PCR and bilateral medial temporal involvement on imaging, which confirms the diagnosis of HHV-6 encephalitis [1,4].

The most common neurological complication of HHV-6 infection is febrile convulsions [8]. However, HHV-6 encephalitis usually affects immunocompromised patients and rarely develops in healthy patients like our patient, while ANEC and ADEM mostly affect healthy patients [2]. The sudden onset and rapid progression of our patient’s illness with thalami involvement support the diagnosis of ANEC, especially in the presence of resolution of restriction diffusion and the development of infarcts in repeated MRI [5]. The elevated CSF protein level can be explained by increased blood-brain barrier permeability or damage to brain parenchyma [9]. The presence of high CSF protein in the absence of CSF pleocytosis supports the diagnosis of both HHV-6 encephalitis and ANEC, but they are against ADEM [10].

Any patient with acute encephalopathy with positive CSF HHV-6 PCR could have HHV-6 encephalitis alone or primary HHV-6 infection with complicated acute necrotizing encephalopathy [1,11]. There are a few cases of pediatric and adult patients with primary HHV-6 infection predisposing to acute necrotizing encephalopathy [11]. Our patient has a mild elevation of his liver enzymes with preserved ammonia, which can also support the diagnosis of ANEC. Patients with ANEC could have liver dysfunction ranging from mild to severe [10]. The neurological sequelae rate has the highest outcome rate in patients with ANEC [12]. They also have a high mortality rate (up to 30%) and a low complete recovery rate [6,12].

If our patient has ANEC, we can estimate his outcome by categorizing him as "high-risk ANEC" by using "the severity score for acute necrotizing encephalopathy (SS-ANEC)" [9]. Our patient has a negative family history of encephalopathy, and no pathogenic variant was identified in the RAN-binding protein 2 (*RANBP2*) gene, which does not exclude ANEC but can make familial ANEC less likely, which has a tendency for recurrence. If no pathogenic variant was identified in the *RANBP2* gene and the patient has evidence of recurrent or familial ANEC, this may indicate that he carries another undiscovered gene mutation in the *RANBP2* gene or another gene not reported previously that is associated with a more severe form of familial ANEC [13].

This patient has asymmetrical involvement in some affected areas, mainly the thalami; that is why we cannot say this patient does not have ADEM or an ADEM-like picture [7]. Remarkably, almost 50% of patients with ADEM have positive anti-MOG antibodies, which were negative in our patient [7]. Our patient’s response to glucocorticoid therapy was not as expected as that of patients with ADEM; they usually respond within days of initiating the treatment, and most of them return to their normal neurological status with complete recovery [7]. ADEM can also develop secondary to primary HHV-6 infection, as reported in pediatric patients by Kamei et al. and in adult patients by Novoa et al. [14,15]. Those associations need to be confirmed in further studies or case series.

The decision to use dual antiviral therapy for HHV-6 encephalitis is controversial. In our case, it depended on several factors, such as the severity of the infection (severe encephalitis), the patient's age, unknown immune status and HIV screening at the initial presentation, the presence of a very high viral load, as well as a suboptimal response to treatment with suspicion of resistance to Ganciclovir. As a result, we think it may be necessary to maintain dual antiviral therapy until we have the full final picture, including the results of sensitivity [16].

## Conclusions

HHV-6 encephalitis rarely affects healthy children, and if it does, we need to rule out complicated forms of encephalitis. There are few reported cases describing the ability of HHV-6 infection to induce acute necrotizing encephalopathy and acute disseminated encephalomyelitis, but this is a rare case of HHV-6 confirmed by elevated CSF protein, positive CSF HHV-6 PCR, and significantly high CSF quantitative PCR, which affect the CNS system and induce ANEC in addition to radiological features of ADEM-like picture at the same time.
